# Supplementary material for: Activation of Epstein–Barr Virus’ Lytic Cycle in Nasopharyngeal Carcinoma Cells by NEO212, a Conjugate of Perillyl Alcohol and Temozolomide
Source: Cancers (Basel). 2024 Feb 26;16(5):936. doi: 10.3390/cancers16050936 (PMC10931041; doi:10.3390/cancers16050936)

Figure S1: Uncropped blots of the Western blots used for Figure 3.

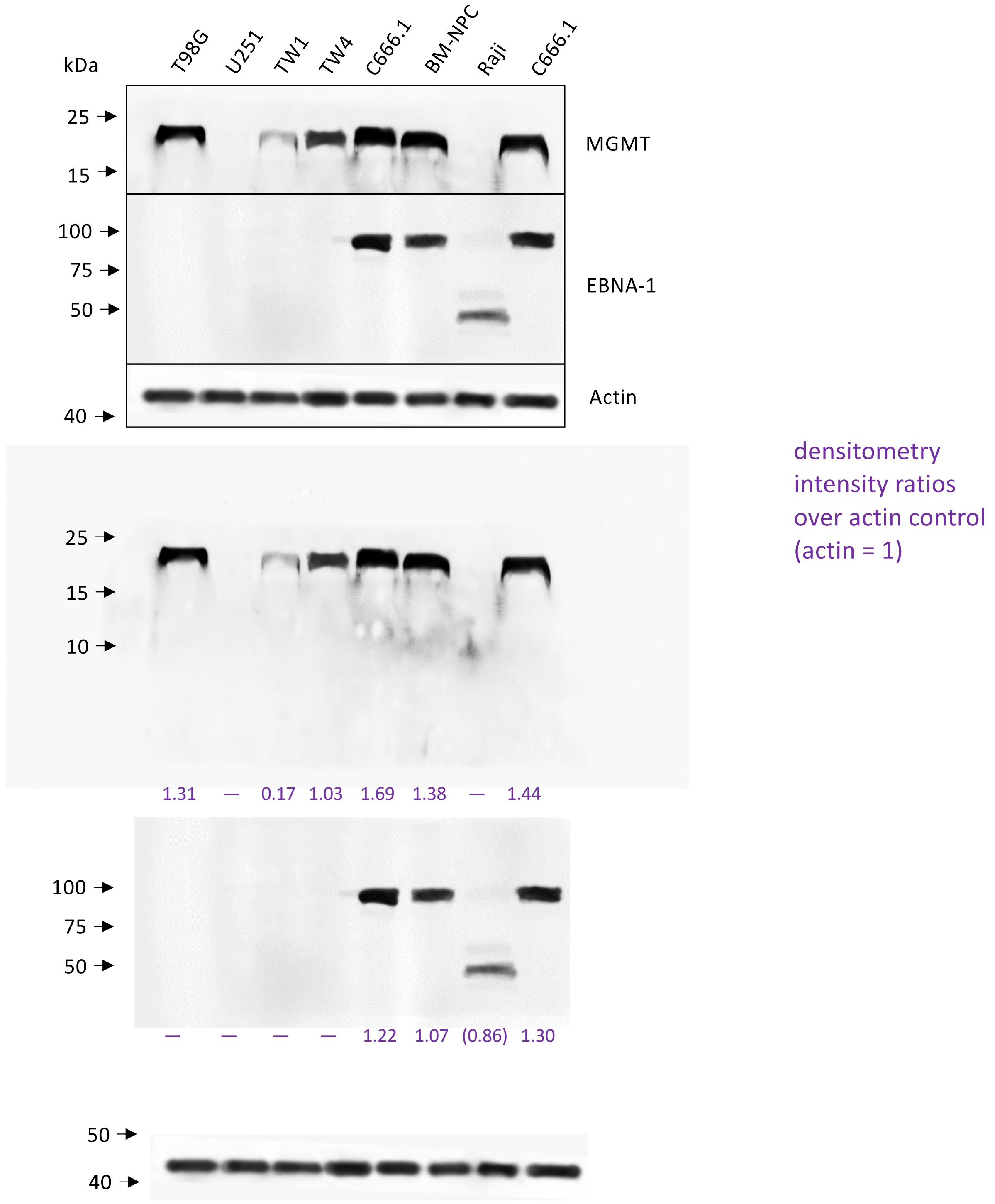

Figure S2: Uncropped blots of the Western blots used for Figure 4A.

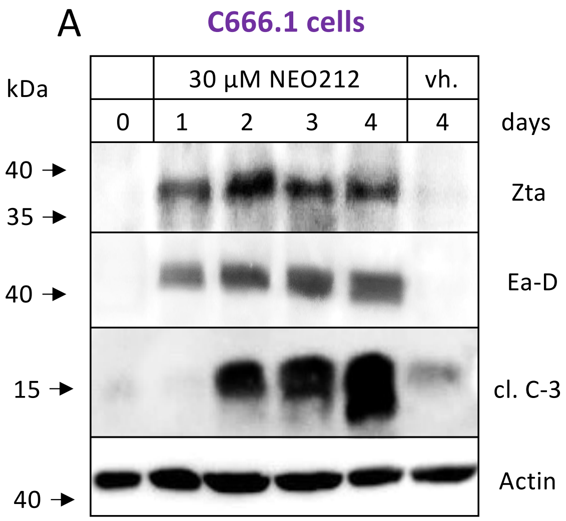

densitometry intensity ratios over actin control (actin = 1)

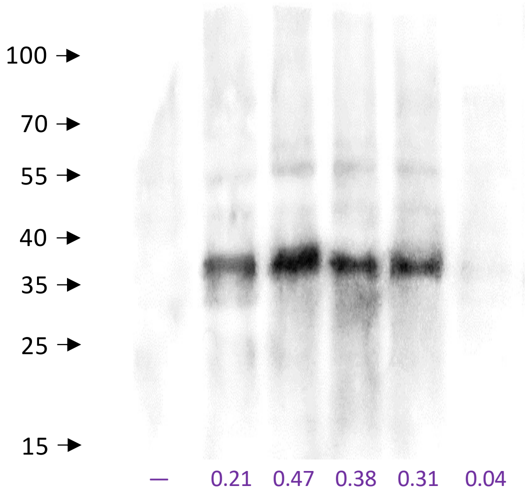

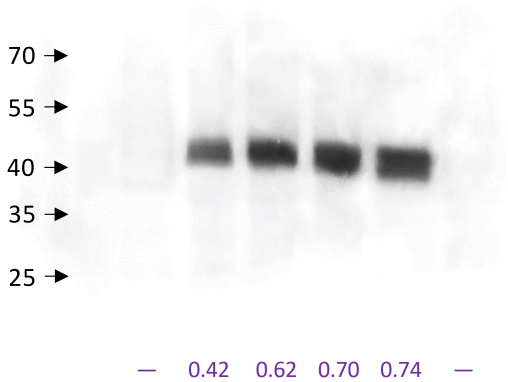

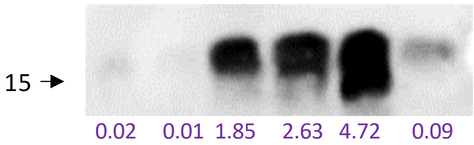

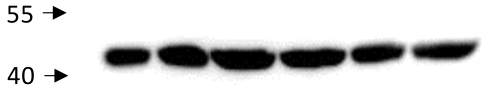

Figure S3: Uncropped blots of the Western blots used for Figure 4B.

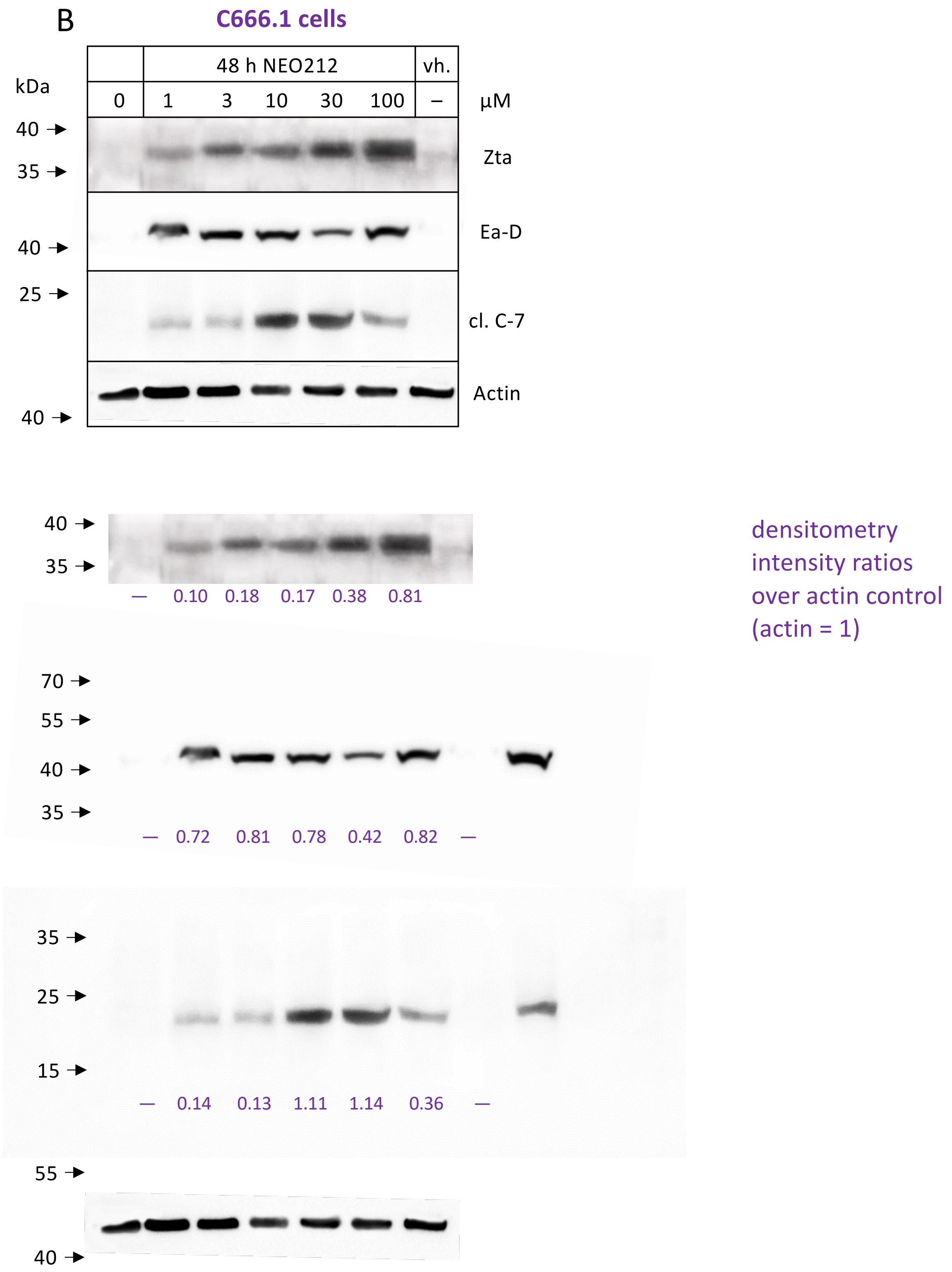

Figure S4: Uncropped blots of the Western blots used for Figure 4C.

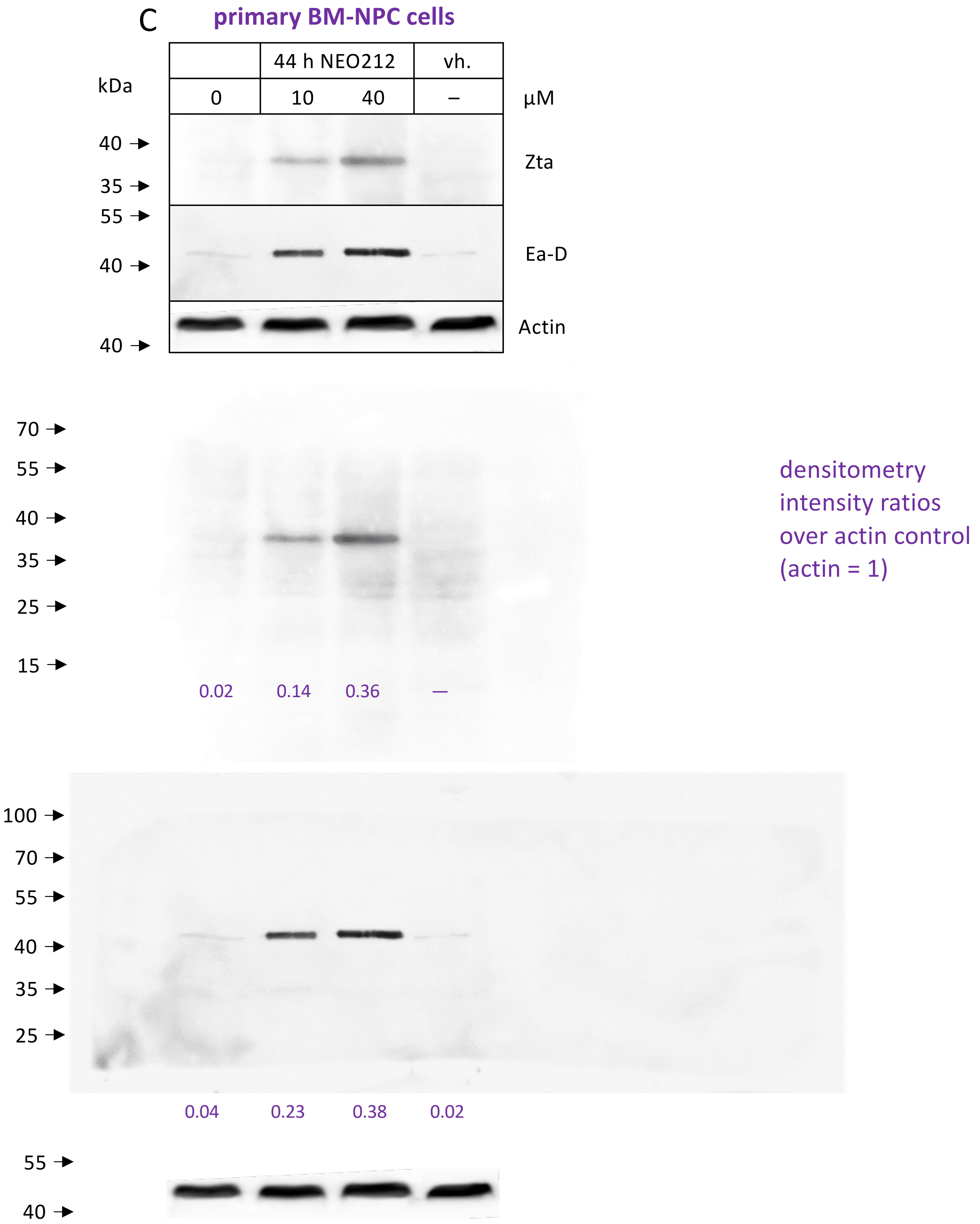

Figure S5: Uncropped blots of the Western blots used for Figure 4D.

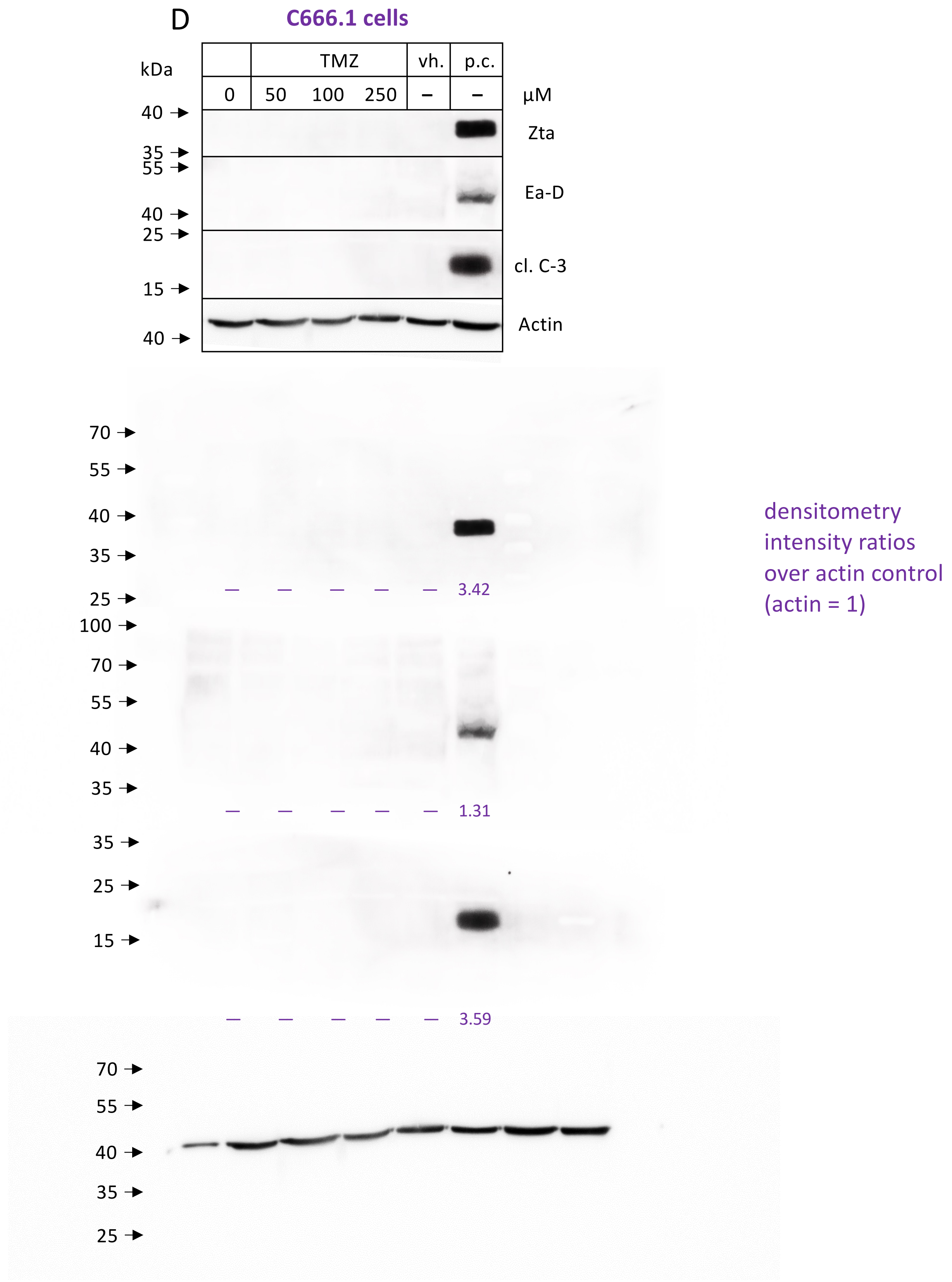

Figure S6: Uncropped blots of the Western blots used for Figure 4E.

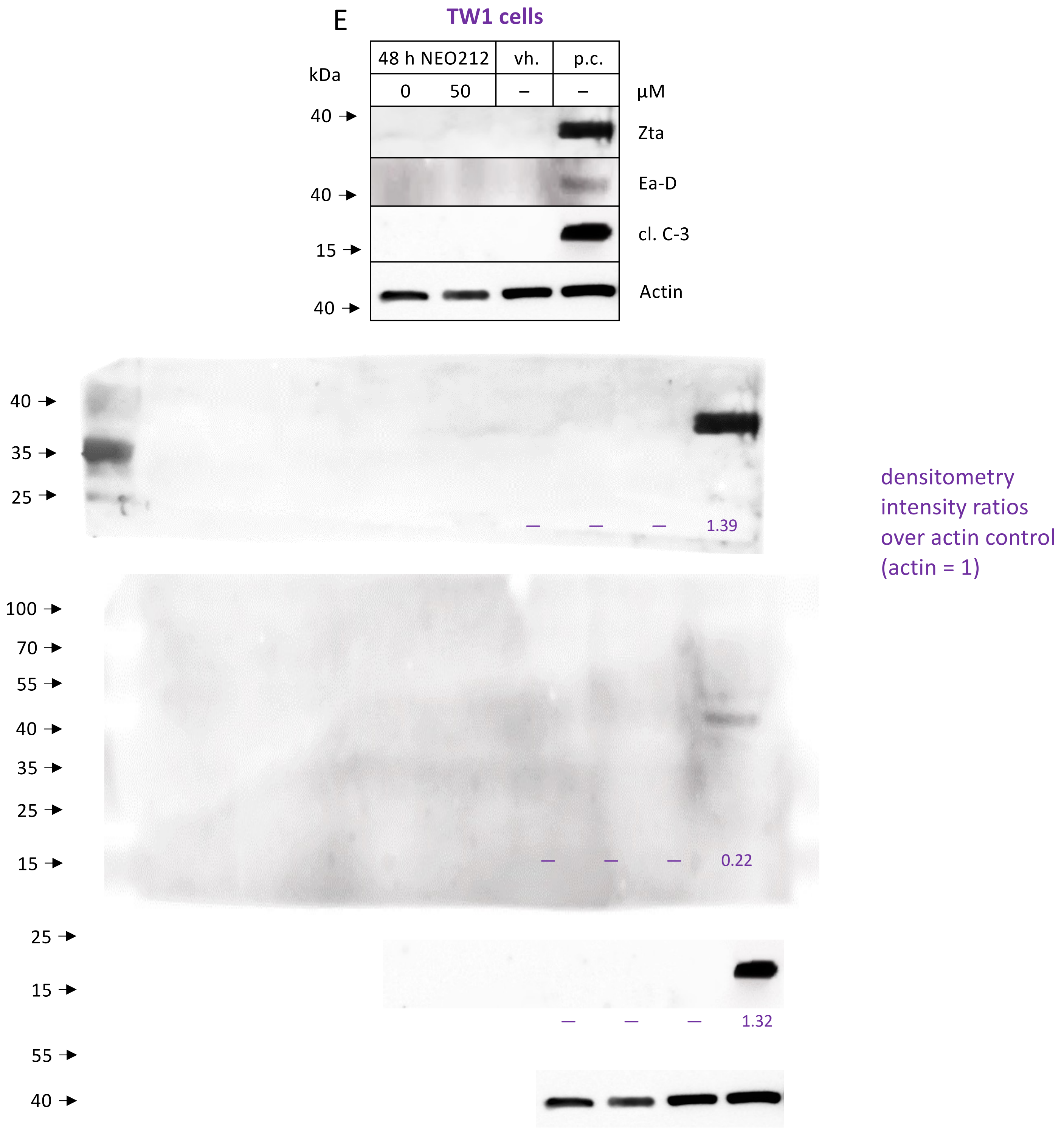

Figure S7: Uncropped blots of the Western blots used for Figure 6.

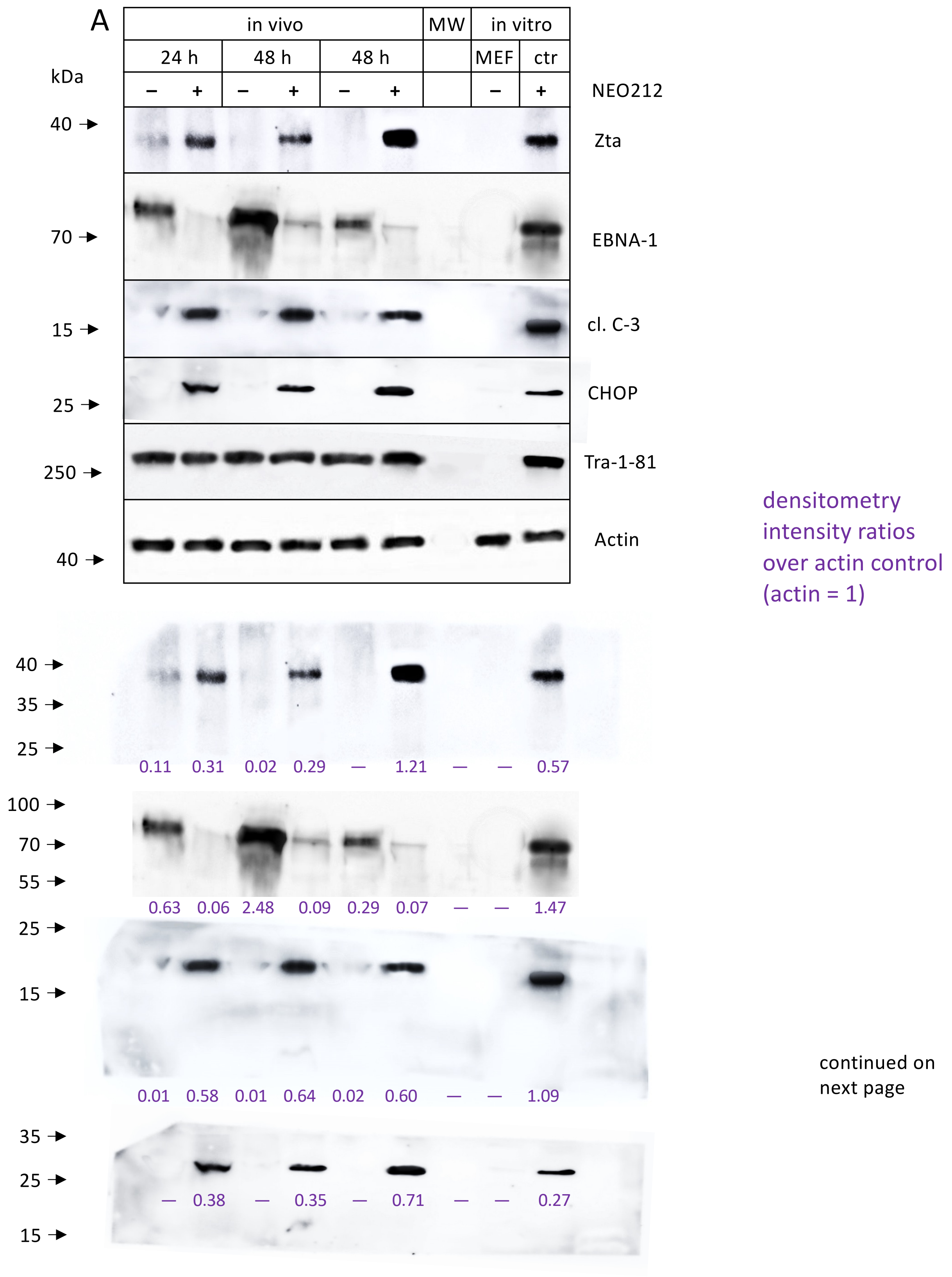

continued on next page

Figure S7 (continued): Uncropped blots of the Western blots used for Figure 6.

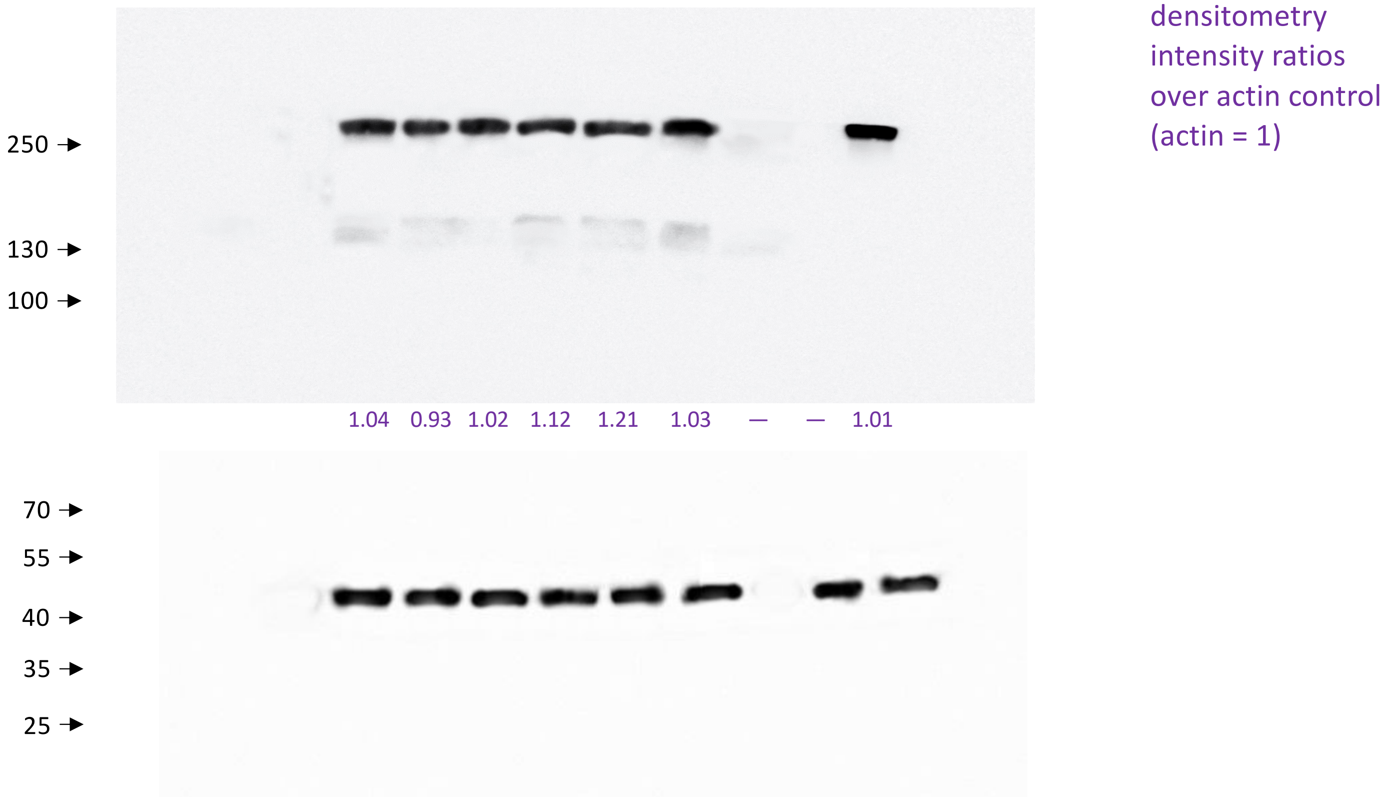

Figure S8: Uncropped blots of the Western blots used for Figure 6.

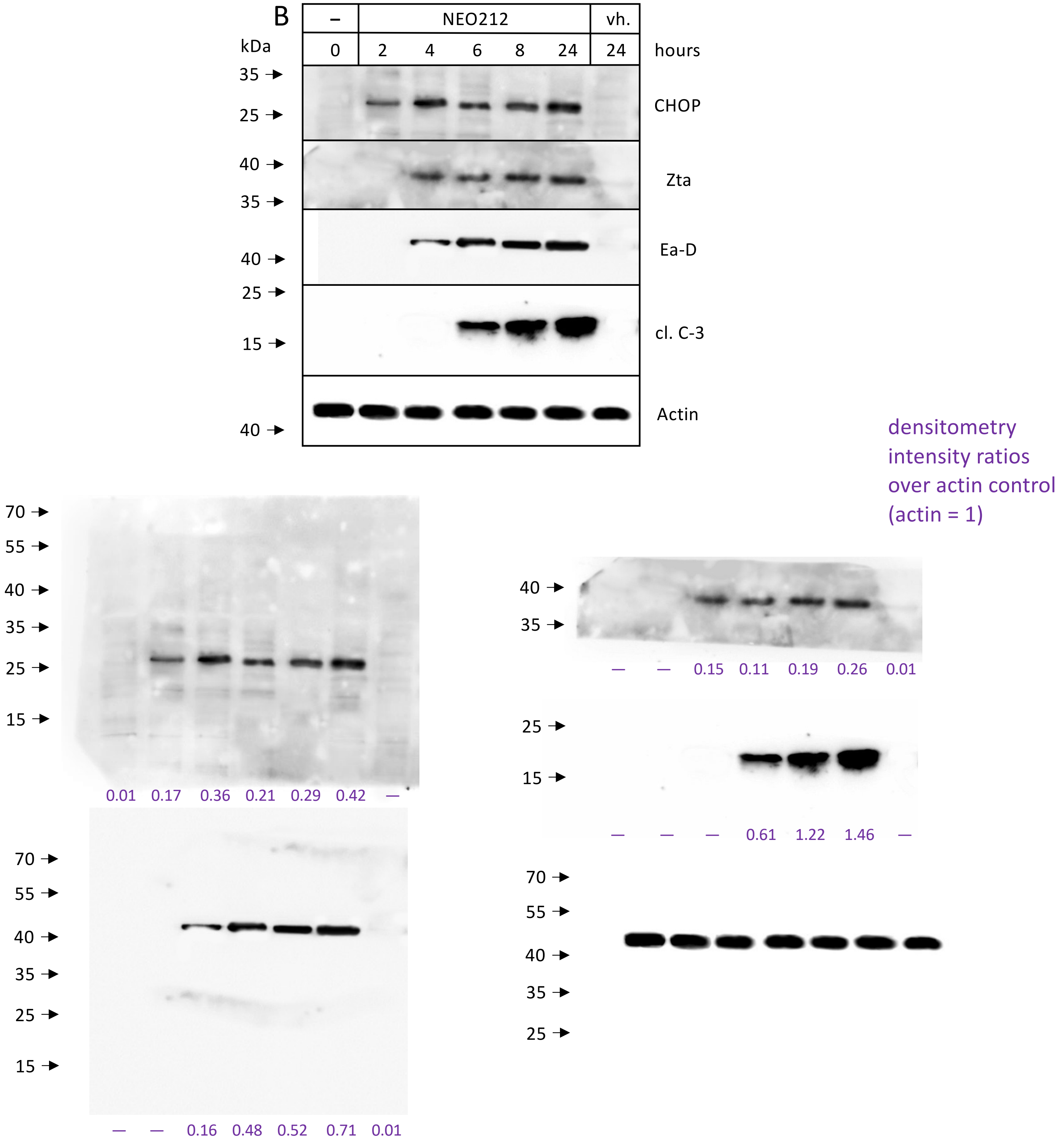

Figure S9: Uncropped blots of the Western blots used for Figure 7.

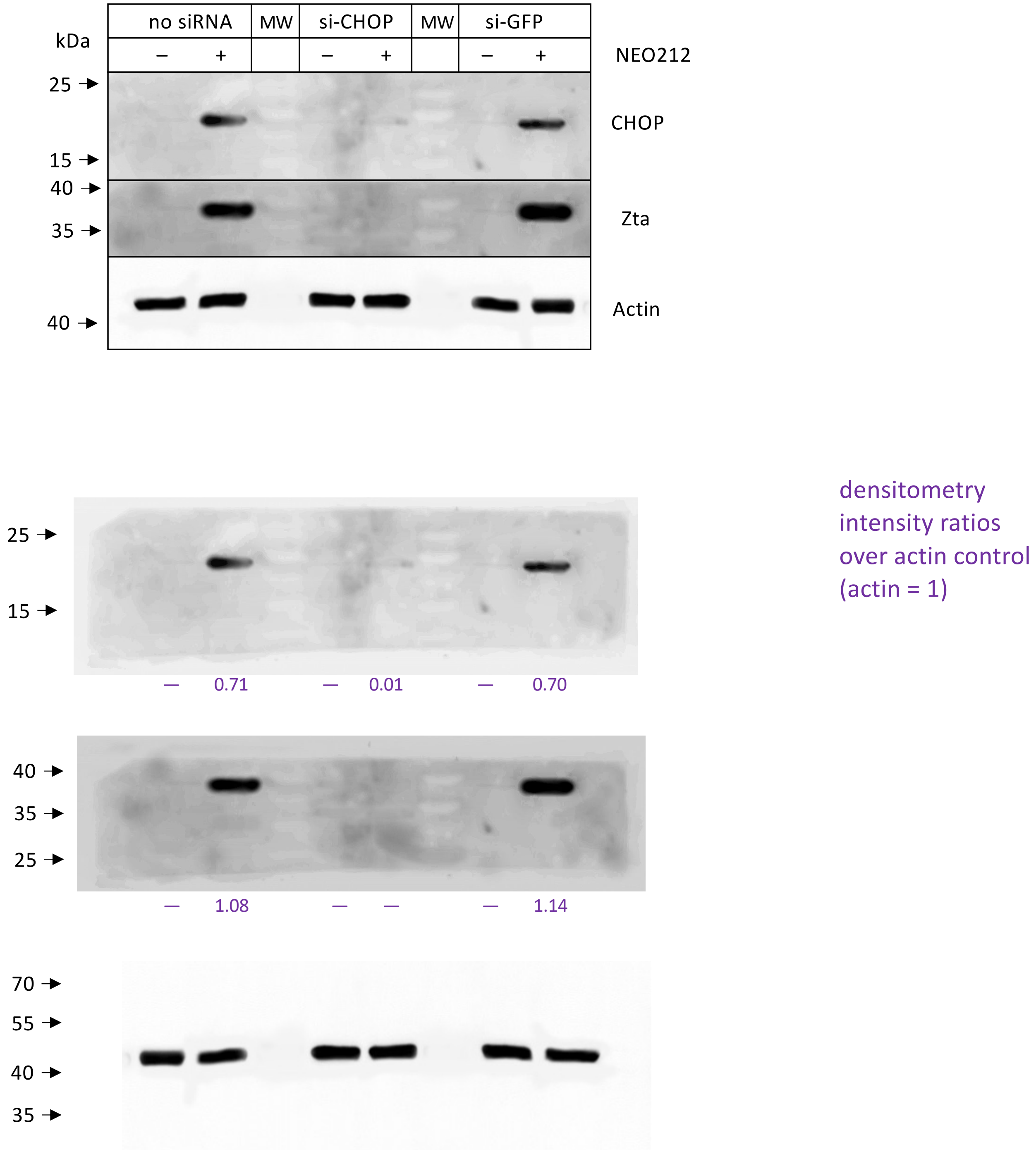

Figure S10: Uncropped blots of the Western blots used for Figure 8.

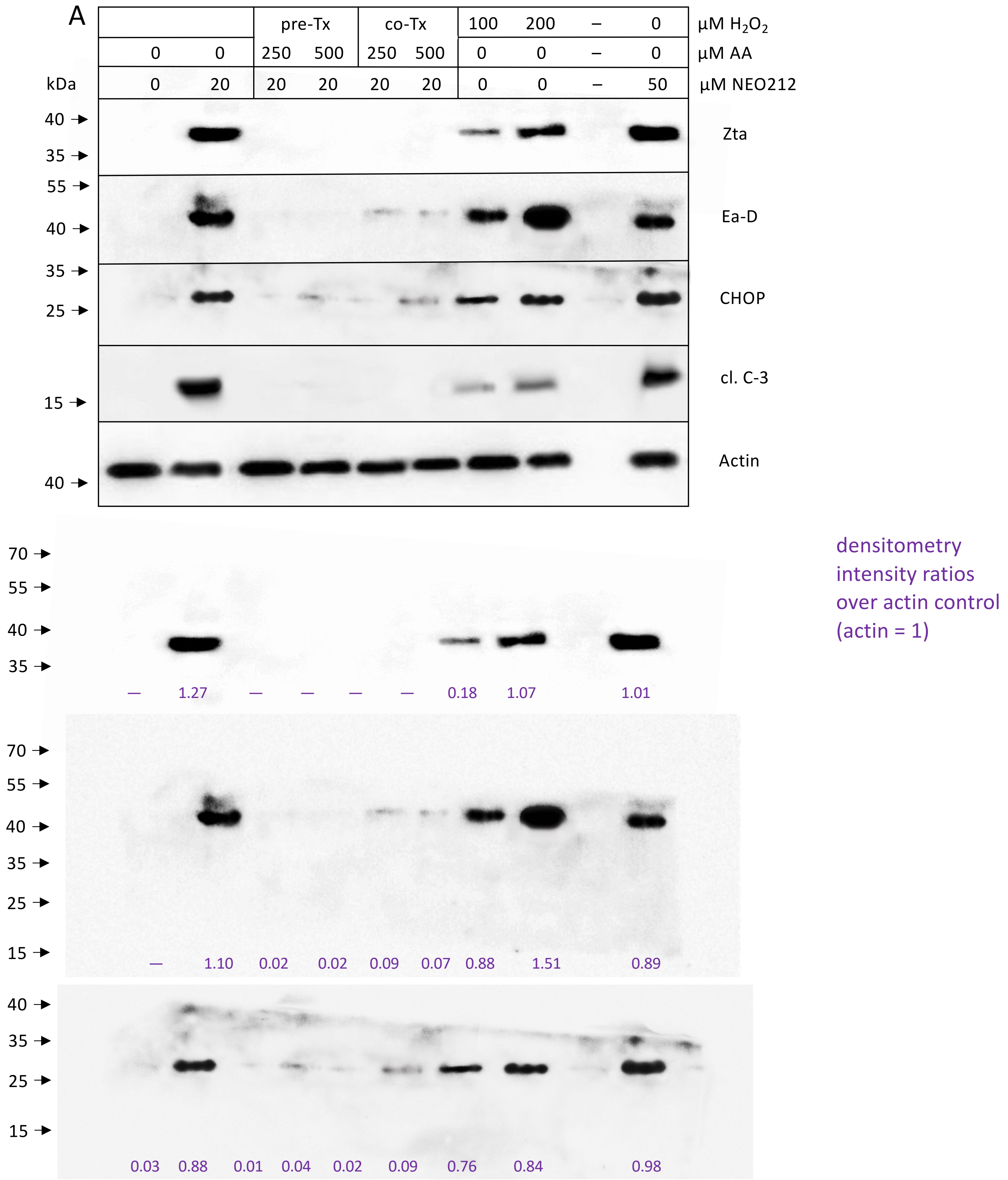

Figure S11: Uncropped blots of the Western blots used for Figure 8.

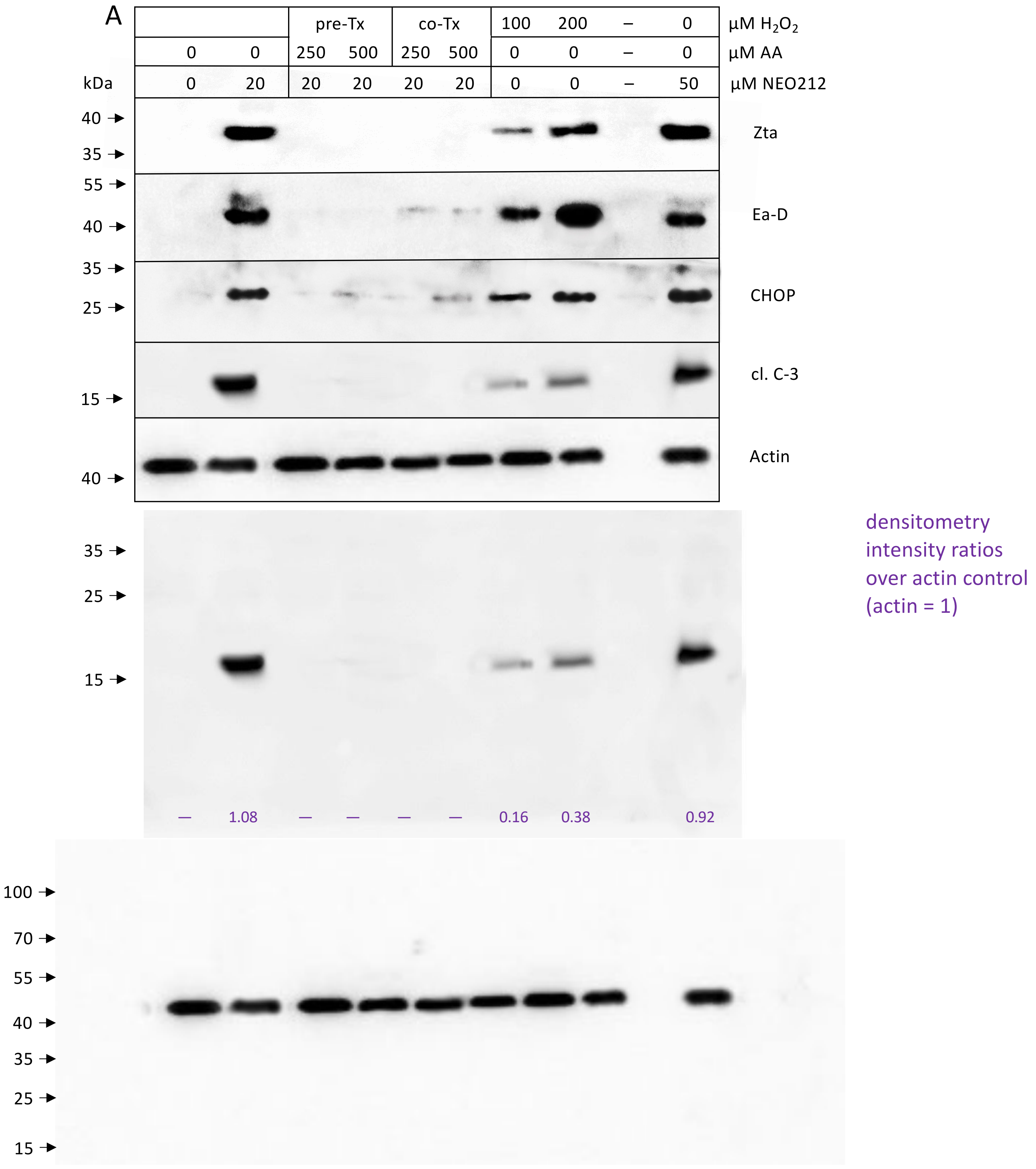

Figure S12: Uncropped blots of the Western blots used for Figure 8.

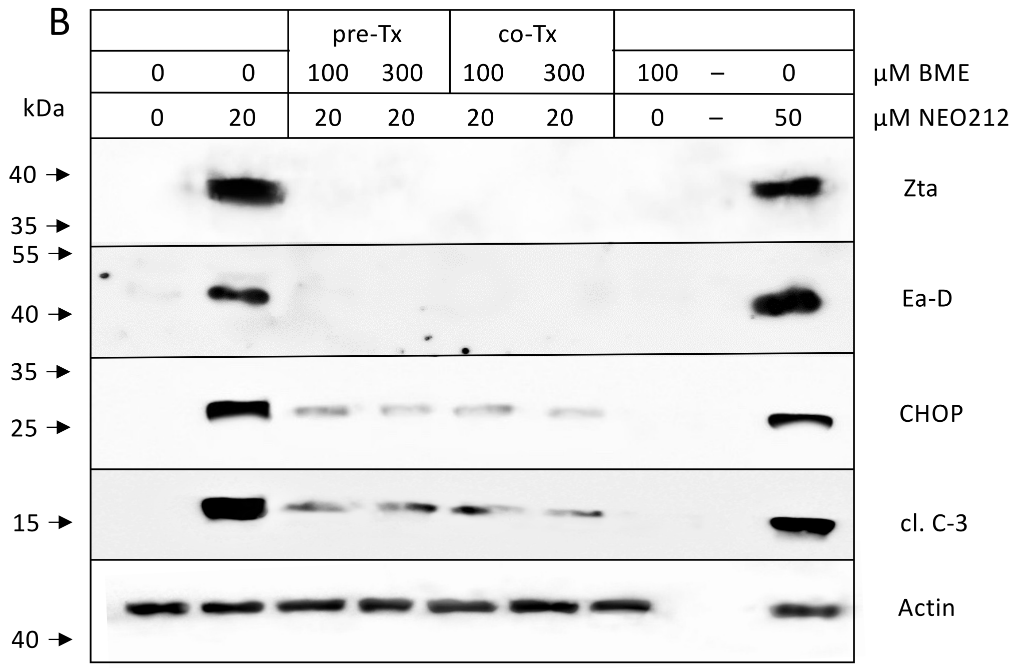

densitometry intensity ratios over actin control (actin = 1)

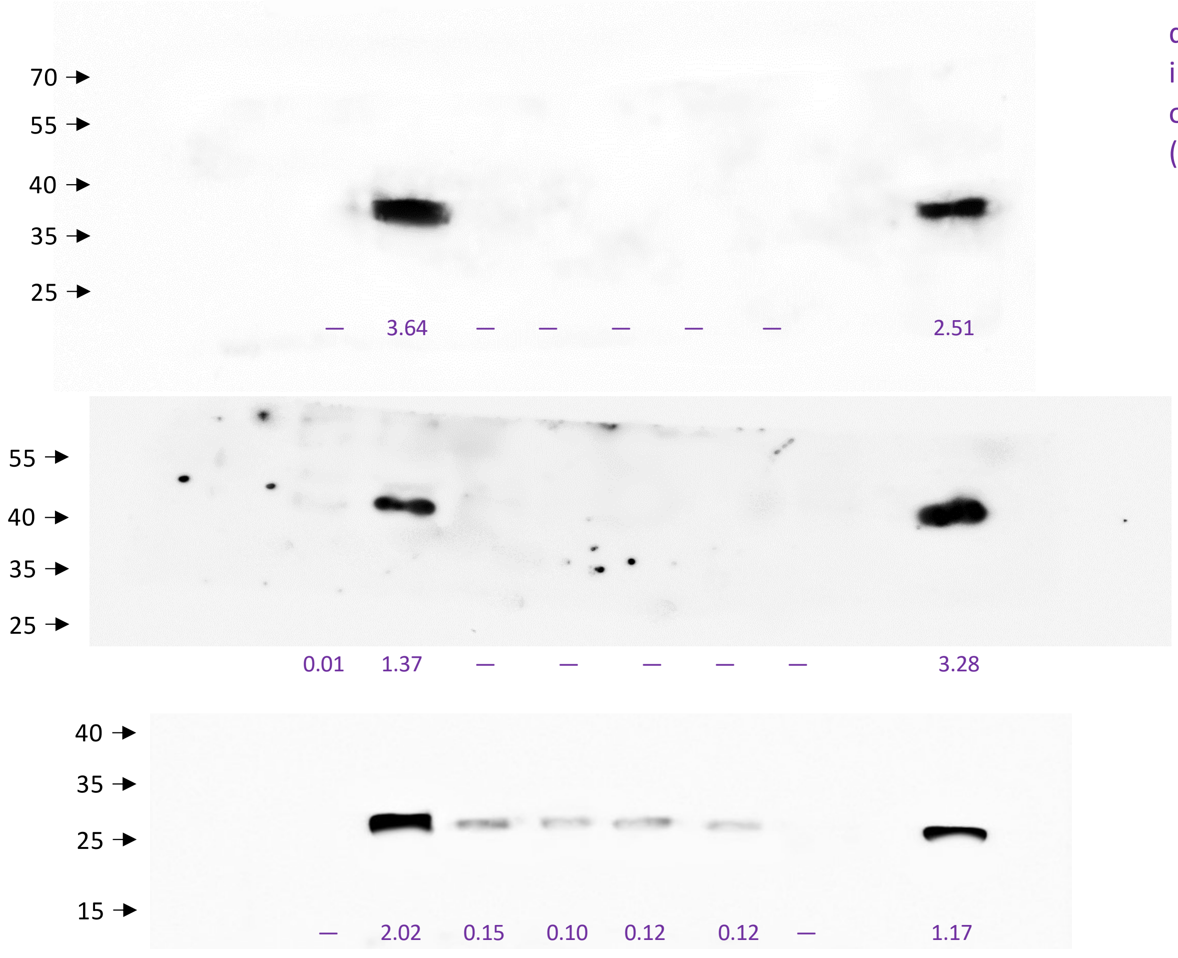

continued on next page

Figure S12 (continued): Uncropped blots of the Western blots used for Figure 8.

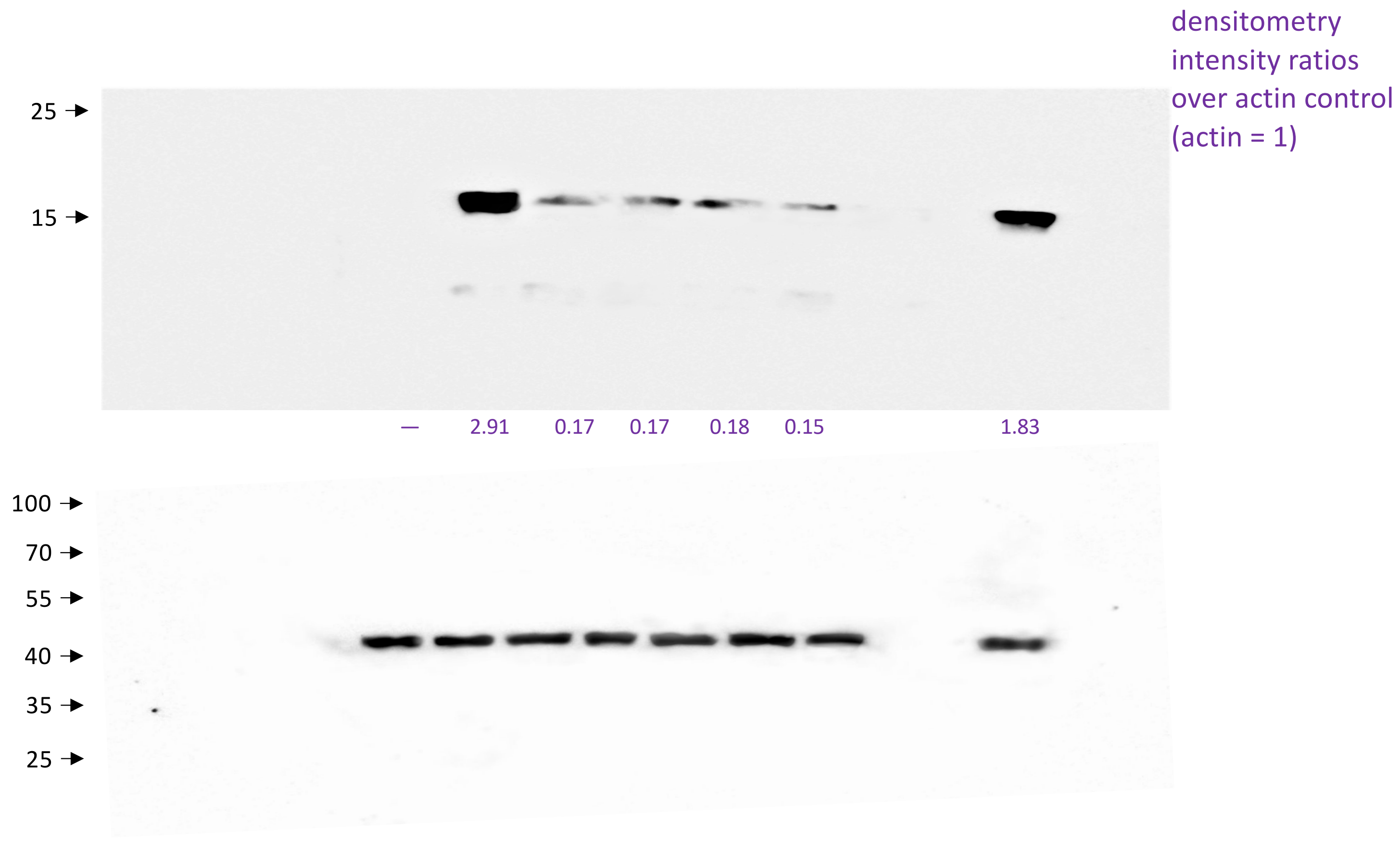

Figure S13: Uncropped blots of the Western blots used for Figure 9.

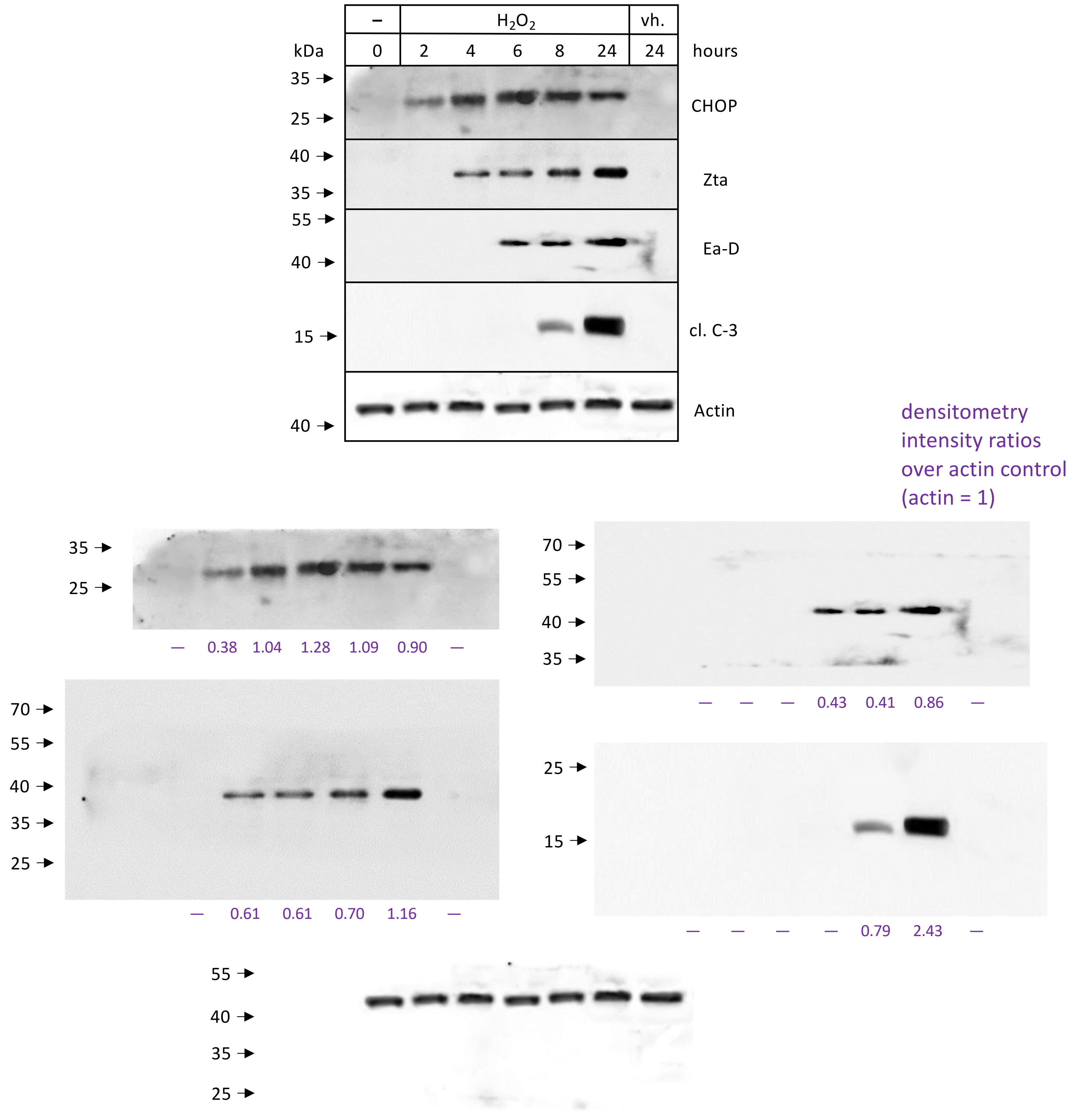

Supplement: Supplementary file 1 [file cancers-16-00936-s001.zip › cancers-2836280 Supplementary Material.pdf]
